# Pharmacologic interventions for the treatment of equine herpesvirus‐1 in domesticated horses: A systematic review

**DOI:** 10.1111/jvim.17016

**Published:** 2024-02-21

**Authors:** Lutz Goehring, David C. Dorman, Klaus Osterrieder, Brandy A. Burgess, Kelsie Dougherty, Peggy Gross, Claire Neinast, Nicola Pusterla, Gisela Soboll‐Hussey, David P. Lunn

**Affiliations:** ^1^ University of Kentucky, College of Agriculture, Food and Environment, Maxwell H. Gluck Equine Research Center, 1400 Nicholasville Road Lexington, Kentucky 40546‐0099 USA; ^2^ College of Veterinary Medicine, North Carolina State University, 1060 William Moore Drive Raleigh, North Carolina 27607 USA; ^3^ Institut für Virologie, Freie Universität Berlin, Robert‐von‐Ostertag‐Str. 7 14163 Berlin Germany; ^4^ College of Veterinary Medicine University of Georgia, 2200 College Station Road Athens, Georgia 30602 USA; ^5^ School of Veterinary Medicine, University of California, Davis, One Garrod Drive Davis, California 95616 USA; ^6^ College of Veterinary Medicine, Michigan State University, Veterinary Medical Center, Room G331, 784 Wilson Road East Lansing, Michigan 48824 USA; ^7^ School of Veterinary Science, University of Liverpool, Leahurst Campus, Chester High Road Neston CH64 7TE United Kingdom

**Keywords:** chemotherapy, equine, equine herpesvirus myeloencephalopathy, herpesvirus‐1

## Abstract

**Background:**

Equine herpes virus type 1 (EHV‐1) infection in horses is associated with upper respiratory disease, neurological disease, abortions, and neonatal death.

**Review Question:**

Does pharmacological therapy decrease either the incidence or severity of disease or infection caused by EHV‐1 in domesticated horses?

**Methods:**

A systematic review was preformed searching AGRICOLA, CAB Abstracts, Cochrane, PubMed, Web of Science, and WHO Global Health Index Medicus Regional Databases to identify articles published before February 15, 2021. Selection criteria were original research reports published in peer reviewed journals, and studies investigating in vivo use of therapeutic agents for prevention or treatment of EHV‐1 in horses. Outcomes assessed included measures related to clinical outcomes that reflect symptomatic EHV‐1 infection or virus infection. We evaluated risk of bias and performed a GRADE evaluation of the quality of evidence for interventions.

**Results:**

A total of 7009 unique studies were identified, of which 9 met the inclusion criteria. Two studies evaluated valacyclovir or small interfering RNAs, and single studies evaluated the use of a *Parapoxvirus ovis*‐based immunomodulator, human alpha interferon, an herbal supplement, a cytosine analog, and heparin. The level of evidence ranged between randomized controlled studies and observational trials. The risk of bias was moderate to high and sample sizes were small. Most studies reported either no benefit or minimal efficacy of the intervention tested.

**Conclusions and Clinical Importance:**

Our review indicates minimal or limited benefit either as a prophylactic or post‐exposure treatment for any of the studied interventions in the mitigation of EHV‐1‐associated disease outcome.

**Abbreviations**
EHM: equine herpesvirus‐1 myeloencephalopathy
EHV‐1: equine herpesvirus‐1
GRADE: Grading of Recommendations, Assessment, Development, and Evaluation
NSAIDs: nonsteroidal anti‐inflammatory drugs
PICO: Population, Intervention, Comparator, and Outcome
RCTs: randomized clinical trials

## 1 INTRODUCTION

Equine herpesvirus‐1 (EHV‐1) is a highly prevalent member of the *Alphaherpesvirinae* that infects horses worldwide.^1^ The virus is transmitted via direct horse‐to‐horse contact via oronasal secretions as well as from contact with contaminated aborted fetuses, placenta, and fomites.^2^, ^3^ Many horses (at least 10%‐30%) are latently infected leading to reactivating infections.^4^, ^5^, ^6^

A biphasic fever response is typically observed in EHV‐1 infected horses with a first peak 1 to 2 days after the initial infection, and a second peak 5 to 7 post‐infection.^5^ EHV‐1 is associated with syndromes including equine herpesvirus‐1 myeloencephalopathy (EHM), respiratory disease in young horses, late term abortion, and neonatal death.^7^, ^8^, ^9^

Control of EHV‐1 relies on a combination of vaccination, infection control, and management practices. Symptomatic horses are treated with supportive care and using compounds that reduce the inflammatory response that underlies EHV‐1‐induced vasculitis.^10^ Anti‐inflammatory drugs including acetylsalicylic acid, flunixin meglumine, dexamethasone, and prednisolone as well as free‐radical scavengers including vitamin E or dimethyl sulphoxide have been recommended.^7^, ^10^, ^11^ The use of anti‐herpesvirus drugs including acyclovir and valacyclovir have also been reported.^12^, ^13^, ^14^ Some therapeutics have also been evaluated in vivo in rodents infected with EHV‐1.^15^, ^16^, ^17^ Despite a number of publications, the scientific basis for the use of pharmacologic agents in the prevention or treatment of EHV‐1 infection in domestic horses is poorly established. Therefore, the goal of this study was to complete a systematic review of the scientific literature to determine whether pharmacological therapy with antivirals, nonsteroidal anti‐inflammatory drugs (NSAIDs), corticosteroids, anti‐coagulants, or other therapies decrease either the incidence or severity of disease or infection caused by EHV‐1 in domesticated horses?

## 2 MATERIALS AND METHODS

### 2.1 Problem formulation and protocol development

A systematic review study protocol was developed using guidelines provided by the Cochrane collaboration.^18^ The protocol detailed the research question, outcomes of interest, outlined a search strategy and the process of data extraction and provided criteria for rating the quality of evidence (Supplementary Item 1). The specific review question and Population, Intervention, Comparator, and Outcome (PICO) statement for the systematic review are as follows:

- Review question: Does pharmacological therapy with antivirals, nonsteroidal anti‐inflammatory drugs (NSAIDs), corticosteroids, anti‐coagulants, or other therapies decrease either the incidence or severity of disease and infection of EHV‐1 in domesticated horses?
- Population: Domesticated horses (*Equus caballus*) without sex, age, or breed restrictions
- Intervention: Any drug therapy, irrespective of dose, route of administration, or duration of treatment, given at any of the following points:
  - Prophylactic treatment in advance of EHV‐1 infection
  - Treatment post‐infection EHV‐1, in the absence of signs of neurological disease
  - Treatment post‐infection EHV‐1, in the presence of signs of neurological disease (EHM: ataxia and weakness)
- Comparator: Horses infected or exposed to EHV‐1 infection, and treated with any drug treatment, compared to placebo‐treated horses, or other dosage of the same treatment (dose response).
- Outcome: All clinical outcomes that reflect symptomatic EHV‐1 infection or viral infection. Presence and degree of viral infection. Clinical outcomes of interest included:
  - Rhinopneumonitis: pyrexia with signs of respiratory disease, including oculo‐nasal discharge, elevated respiratory rate, cough, lethargy
  - Abortion in the third trimester
  - Equine herpesvirus myeloencephalopathy (EHM)
  - Neonatal infection
  - Ocular disease
  - Male reproductive tract infection (eg, orchitis)

### 2.2 Study selection

Studies included in the systematic review were not restricted by either publication date or language. Only peer‐reviewed articles were considered for inclusion. Studies included in the review were randomized clinical trials (RCTs), non‐randomized intervention trials, and observational studies. The following inclusion and exclusion criteria were used to select studies:

- Inclusion:
  - Domesticated equids without sex, age, breed, or immunological status restriction.
  - Therapeutic trials that evaluated the efficacy of a drug against EHV‐1 experimental or natural infection.
  - Studies that used a placebo or other dosage of same drug.
  - Study included clinical outcomes that reflect symptomatic EHV‐1 infection.
  - Endpoints related to drug efficacy: relative reduction in EHV‐1 disease risk; reduction in odds of EHV‐1 infection.
  - Studies were not excluded based on year, language, or quality.
- Exclusion:
  - Absence of the selected clinical or virological outcomes.
  - Wrong virus species.
  - Lack of concurrent control or comparator.
  - Wrong animal species (not *Equus caballus*).
  - Purely descriptive observational studies.
  - No original data.

### 2.3 Search methods for identification of studies

Searches for relevant existing systematic reviews were performed initially to avoid duplicating any recent work or work in progress. PubMed and the systematic‐review protocol registries PROSPERO and CAMARADES were searched for systematic reviews. No previous relevant systematic reviews were found.

This systematic review followed the PRISMA (Preferred Reporting Items for Systematic Reviews and Meta‐Analyses) statement guidelines. The PubMed search was adapted for the following databases: Web of Science, Cab Abstracts, WHO Global Health Index Medicus Regional Databases, AGRICOLA (AGRICultural OnLine Access), and Cochrane (see Supplementary Item 2). In conducting our search, we used a combination of controlled vocabulary and key words for the following concepts: (a) EHV‐1, (b) horses, and (c) pharmacological therapies. We did not seek to identify research abstracts from meeting proceedings or unpublished studies because these are not commonly subjected to exhaustive peer review. We did not limit to language or publication date. References from included studies were scanned during full text review and did not result in identification of additional studies. All citations were imported into Covidence systematic review software (Veritas Health Innovation, Melbourne, Australia) for peer review by the research team. Titles and abstracts relevant to our study were retrieved and searched for full text. References from included studies were hand‐searched to identify any additional relevant studies for analysis. The literature search was initially conducted on December 18, 2019 and updated on February 15, 2021. Literature searches were performed by a medical librarian and coauthor (Peggy Gross) with experience in the conduct of systematic reviews.

Retrieved references were independently screened at the title and abstract level and at the full‐text level for adherence to the PICO statement by 2 people (David C. Dorman and Lutz Goehring) using Covidence software. At the title and abstract screening level, if there was disagreement between the reviewers or an abstract was not available, the reference was passed on to the full‐text screening level. At the full‐text level, disagreements about whether to include a reference were discussed by the 2 reviewers (David C. Dorman and Lutz Goehring) to reach agreement; if consensus was not reached, then a third team member (David P. Lunn) resolved the differences. Coauthors of studies were excluded from evaluating their publications for inclusion or exclusion. One author (David P. Lunn) of this systematic review was involved in the design, execution, and reporting of 3 of the included studies. This author was not involved in the evaluation of these studies or the primary author of this review.

### 2.4 Data extraction

One author (David C. Dorman) performed data extraction using a customized data‐extraction form, and 2 other authors (Kelsie Dougherty and Claire Neinast) verified the records for accuracy and completeness. Disagreements were resolved by discussion among the data extraction team. The data items extracted included study design, funding source, characteristics of trial participants (number and breed of horses examined), intervention characteristics (dose, route of administration and timing of administration), viral challenge (dose, route of administration, and timing of administration), the type of control group used, outcomes measured, and study results.

### 2.5 Methods of review

Risk of bias in individual studies was assessed by 3 authors (David C. Dorman, Kelsie Dougherty, and Claire Neinast) working in pairs independently of each other using the Covidence systematic review software. Coauthors of studies were excluded from evaluating their publications for risk of bias. Each member of this 3‐person team evaluated each study according to prespecified criteria developed for animal experiments.^19^ The risk‐of‐bias domains used in this study included: generation of allocation sequence; similarity of groups at baseline, concealment of animal allocation to groups, animals randomly pastured or housed, blinding of participants and personnel to the intervention; random selection of animals for outcome assessment, blinding of outcome assessment; incomplete outcome data; selective reporting; and other sources of bias. Available risk of bias ratings for each domain were: low risk of bias; unknown risk of bias; or high risk of bias. Each individual study was assessed using the signaling questions and other guidance provided in the SYRCLE tool.^19^ These signaling questions provide guidance on conditions that meet the domain criteria resulting in a low risk of bias and other conditions that would not meet the criteria resulting in a no response resulting in a high risk of bias. Information or study procedures that were not reported were assumed not to have been conducted, resulting in an assessment of “unknown” risk of bias. Study authors were not contacted for missing data. Because the number of retrieved studies was limited no attempt was made to assess publication bias. Risk of bias assessments were considered in the subsequent step (method of analysis and evidence synthesis).

### 2.6 Method of analysis and evidence synthesis

A narrative review was performed of all studies. Assessment of the quality, quantity and consistency of evidence across studies was also independently performed by 2 authors (blinded for review) using the Grading of Recommendations, Assessment, Development, and Evaluation (GRADE) approach.^20^ Separate teams were used to screen the literature and a second team composed of individuals with no ties to the published studies were used to extract data, perform risk of bias assessments, and complete the GRADE evaluation of the available literature.

The approach used in this study was adapted from the GRADE approaches previously described by Worrall,^21^ Sullivan,^22^ the United States National Academies of Sciences, Engineering, and Medicine (NASEM),^23^ and the Office of Health Assessment and Translation (OHAT).^24^ In brief, studies on a particular outcome were initially grouped by key study design features, and each grouping of studies was given an initial confidence rating based on those features. An initial confidence rating for the body of evidence for a specific outcome was determined by the ability of the study design to ensure that the exposure preceded the outcome and was associated with the outcome.^23^, ^24^ The 4 features that were used to assess studies were: the exposure was experimentally controlled, exposures occurred before outcome, the outcome was assessed on the individual level, and a comparative group was available.^23^, ^24^ Randomized controlled trials and placebo‐controlled experimental studies included each of these features and were initially ascribed as high‐quality evidence (initial score = ++++). Nonrandomized intervention trials and observational studies were rated as moderate‐quality evidence (initial score = +++). Several factors were then considered to determine whether this initial rating should be either downgraded or upgraded. Factors that could downgrade the rating included quality, indirectness, inconsistency, and imprecision. Factors that could upgrade the rating included large magnitude of effect, dose response, and accounting for plausible confounders.

To obtain the final GRADE confidence rating for a given outcome, the initial confidence rating could be reduced based on criteria related to the following 4 categories: quality, directness, consistency, and precision. Downgrading for quality occurred if there was a collective concern about the overall risk of bias for the studies being considered. The overall risk of bias for each family of studies was summarized as either low risk (low risk of bias in all key criteria; no deduction), moderate risk (serious risk of bias for 1 or more critical risk of bias criterion; 1 or 2 level deduction), or high risk of bias (extreme risk of bias for 1 or more critical risk of bias criterion; 2 level deduction). Downgrades for quality focused on key risk of bias elements which included blinding of investigators and outcome assessors to treatment groups, similarity of subjects at the start of the study, and risk of bias concerns including statistical analyses or an unspecified role of study sponsors. Evaluation of the study for indirectness considered the following factors: population differences, differences in interventions (applicability), and use of surrogate outcomes or insufficient timeframe.^23^, ^24^ This downgrade was not used in this review. We also downgraded the rating of the quality of the evidence if the intervention cannot be implemented by equine practitioners with the same rigor or technical sophistication as was present in the study evaluating the therapeutic. Since all interventions could be implemented, this downgrade was not used in this review. Studies were also downgraded for inconsistency.^23^, ^24^ This downgrade considered the similarity of results across studies. For example, no downgrade occurred if the studies that contributed the most to the effect estimate had consistent results, or inconsistent results were explained satisfactorily. A reduction in the initial confidence rating occurred if 1 or more studies that contributed the most to the effect estimate had moderate to serious inconsistency in their results that went unexplained by subsequent analyses. A statistically based approach to assess inconsistency or heterogenicity was not performed. Instead, we relied on qualitative analysis of the degree of variability in point estimates (eg, a downgrade for inconsistency could be applied if mixed, negative and positive results were reported) and when available overlap of confidence intervals. Finally, studies could be downgraded for imprecision.^23^, ^24^ Odds ratios with 95% confidence intervals were calculated for the incidence of outcomes of interest using an online calculator (https://www.medcalc.org/calc/odds_ratio.php). No downgrade occurred if the studies that contributed the most to the effect estimate possessed at least 80% power and/or adequate sample size to determine an effect reliably.^22^ A downgrade occurred if 1 or more studies that contributed the most to the effect estimate were underpowered (<80% power) and/or had an inadequate sample size to determine an effect reliably.

The initial rating was upgraded for factors that increased our confidence in the results. Upgrades were applied to observational studies and included large magnitude of effect, dose response, and if all plausible confounders or other biases were accounted for thereby increasing our confidence in the estimated effect.^20^ After a final confidence rating was determined, the rating was translated into a level of evidence. For example, an initial confidence rating of high as in the case of a placebo‐controlled intervention study with 3 of more downgrades was categorized as having a low final confidence level; 2 downgrades resulted in a low confidence level; 1 downgrade resulted in high; and no downgrades would result in a high final confidence level. An upgrade could offset a downgrade resulting in a higher final confidence level. Evidence profiles and summary‐of‐findings tables were created using a customized form.

## 3 RESULTS

### 3.1 Results of the search

**FIGURE 1:**
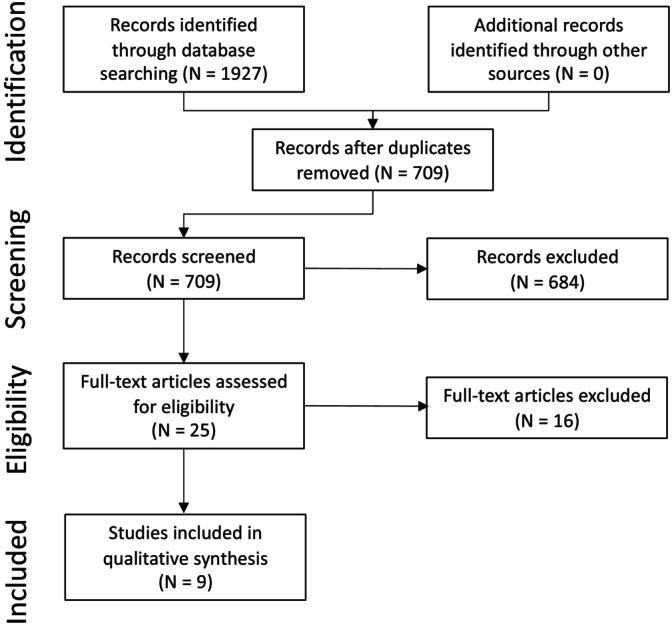
Summary of the search and screening of the literature therapeutic intervention in EHV‐1 infection. Full text articles were excluded for the following reasons: wrong virus or no virus challenge (n = 5); no original data (n = 4), duplicate record (n = 3), wrong animal species (n = 3), or wrong study design (n = 1).

The search strategy identified 1892 citations, of which 1193 were duplicate citations. Another 674 citations were excluded based on the title or abstract. Literature was almost entirely identified and retrieved from electronic bibliographic sources. No studies were identified from hand searching reference lists provided in the studies that met inclusion criteria. A total of 25 studies were assessed for inclusion using a review of the full text. A list of the 16 studies excluded at the full text review stage, with the reason for exclusion, are provided in Supplementary Item 3. Nine studies met the inclusion criteria for this review (Supplementary Item 4), which also includes funding sources for the studies. A flow diagram for inclusion of studies in the systematic review is provided in Figure 1.

### 3.2 Characteristics of included studies

Key study characteristics are provided in Table 1. Eight studies were placebo‐controlled and involved experimental EHV‐1 infection.^12^, ^14^, ^16^, ^25^, ^26^, ^27^, ^28^, ^29^ Neuropathic EHV‐1 strains including rAb4, Ab4, 03P37, Findlay OH 2003 (T953), and G2254/D752 Pol were involved in several included studies.^12^, ^14^, ^16^, ^25^, ^27^, ^30^ One study used the Army 183 strain,^28^ another comingled study horses with other horses that had been infected with the European strain 121412,^26^ while a third study did not identify the strain of virus used in the study.^29^ Three of the studies were considered high‐quality randomized, blinded, placebo‐controlled experimental challenge studies.^14^, ^25^, ^28^ One study was an observational trial involving a naturally occurring EHV‐1 infection.^30^ Pharmacologic agents included valacyclovir,^12^, ^14^ small interfering RNAs,^25^, ^27^ a *Parapoxvirus ovis*‐based immunomodulator,^26^ human alpha interferon,^28^ an herbal supplement,^29^ a cytosine analog,^16^ and heparin.^30^ The herbal supplement included extracts from *Withanis somnifera*, *Ocimum sanctum*, *Emblica officinale*, *Tinospora cardiofolia* and several acylsteryglucosides including sitoindosides VII, and VIII and glcowithanolides including sitoindosides IX and X.^29^ This study^29^ also provided an EHV‐1 vaccine to study horses before viral challenge. Several studies evaluated prophylactic administration of the pharmacologic agent before EHV‐1 infection.^12^, ^14^, ^25^, ^26^, ^27^, ^28^, ^29^ The same studies also continued treatment after the EHV‐1 challenge. Two experimental studies evaluated the efficacy of the pharmacologic agent after EHV‐1 infection.^14^, ^16^ The included studies generally involved small numbers of animals (<20 horses total) and often had wide (>5 years) age ranges (Supplementary Item 5). Breeds included Shetland ponies, Welsh Mountain pony, Light horse breeds, Gypsy Cob, Thoroughbred, Quarter horse, and Kathiawari (Supplementary Item 5). In 2 studies, breeds were not identified.^27^, ^30^ All placebo‐controlled studies included physical and neurologic examinations including measurement of rectal temperature and collection of samples for viral titers using a variety of test methods. Additional information regarding demographics and pre‐study EHV‐1 status of horses in the included studies is provided in Supplementary Item 5.

**TABLE 1:** Key characteristics of included studies.

Treatment	Treatment: Dose, duration, route	Study	Study design	Study comparison	EHV‐1 viral challenge strain, dose, and route	Sample size
Valacyclovir	40 mg/kg bw, TID, for 5 or 7 d, PO. Rx started 1 h before infection	Garré et al. (2009)	Placebo controlled	VAL vs PBO	03P37, 1 × 10^6.5^ TCID_50_, 50% PO, 50% IN	Rx 5 d: 2 Rx 7 d: 2 PBO: 4
	Loading dose = 27 mg/kg, q8h for 2 d, PO. Maintenance dose = 18 mg/kg, q12h, PO. Rx began at either −1 dpi (prophylactic) or to febrile horses, and either continued for 1 or 2 wk.	Maxwell et al. (2017)	Randomized, blinded, and placebo controlled	VAL vs PBO	Findlay OH 2003 (T953), 1 × 10^7^ PFU, IN	Rx 1 wk: 3/group Rx 2 wk: 3 (prophylactic); 2 (febrile) PBO: 6 (total both study arms)
siRNA for gB3 and iOri2	750 pmol sigB3 and 750 pmol siOri2, at −12 and 12 h post infection, IN	Brosnahan et al. (2010)	Randomized, blinded, and placebo controlled	Combined sigB3 and siOri2 vs siLuc	rAb4, 1 × 10^7^ PFU, IN	Rx: 10 PBO: 4
	Rx loading dose: 30 picomole sigB3 and 30 picomole siOri2. Rx maintenance dose: 20 picomole sigB3 and 20 picomole siOri2. PBO loading dose: 60 pmol siLuc. PBO maintenance dose: 40 pmol siLuc. −24, 12, 24, 36, and 48 h after infection. IN	Perkins et al. (2013)	Randomized and placebo controlled	Combined sigB3 and siOri2 vs siLuc	Ab4, 1 × 10^7^ PFU; IN	Rx: 8 PBO: 6
HPMC	1 mg/kg, twice (doi and 3 dpi) or 20 mg/kg, once (doi), SQ	Gibson et al. (1992)	Placebo controlled	HPMPC vs PBO	Ab4, 1 × 10^7^ PFU, IN	Rx low dose: 1 Rx high dose: 1 PBO: 3
iPPVO	Rx: 2 mL. Given −2, 0 and 7 dpi. IM.	Ons et al. (2014)	Randomized, placebo controlled	iPPVO vs PBO	Comingled with six untreated horses inoculated (IN) 3 h prior with 10^5.9^ TCID_50_ strain 121412	Rx: 11 PBO: 12
α‐2a IFN	Low Rx: 0.22 U/kg of body weight, PO, on −2, −1, 0, and 1 dpi. High Rx: 2.2 U/kg, PO, on −2, −1, 0, and 1 dpi.	Seahorn et al. (1990)	Randomized, blinded, and placebo controlled	α‐2a IFN vs PBO	Army 183 1.5 × 10^5^ TCID_50_; IN	Low: 6 High: 6 PBO: 6
Herbal product	25 mg/kg, PO, sid for 30 d starting at −90 dpi.	Verma et al. (1999)	Randomized and placebo controlled	Herbal product^b^ vs PBO	NR 1 × 10^6^ TCID_50_; IN	Rx: 2 PBO: 2
Heparin	25 000 IU, SQ, BID for 3 days. Starting on Day 10 of the outbreak.	Walter et al. (2016)	Observational	Heparin vs no treatment	G2254/D752 Pol	Rx: 31 No Rx: 30

Abbreviations: d, day; doi, day of infection; dpi, day post infection; gB3, glycoprotein B: hours; HPMPC, (S)‐l‐[(3‐hydroxy‐2‐phosphonyl methoxy) propyl] cytosine; IFN, interferon; IN, intranasal; iPPVO, inactivated *Parapoxvirus ovis* (Zylexis^â^); Luc, firefly luciferase; Ori2, origin binding protein helicase; PBO, placebo (control); PFU, plaque‐forming units; pi, post‐infection; PO, per os (oral); q, every; Rx, treatment; si, small interfering; sid, once a day; siRNA, small interfering RNA; SQ, subcutaneous; TCID_50_, tissue culture infectious dose50; TID, three times daily; VAL, valacyclovir; wk, week.

### 3.3 Risk of bias in individual studies

**FIGURE 2:**
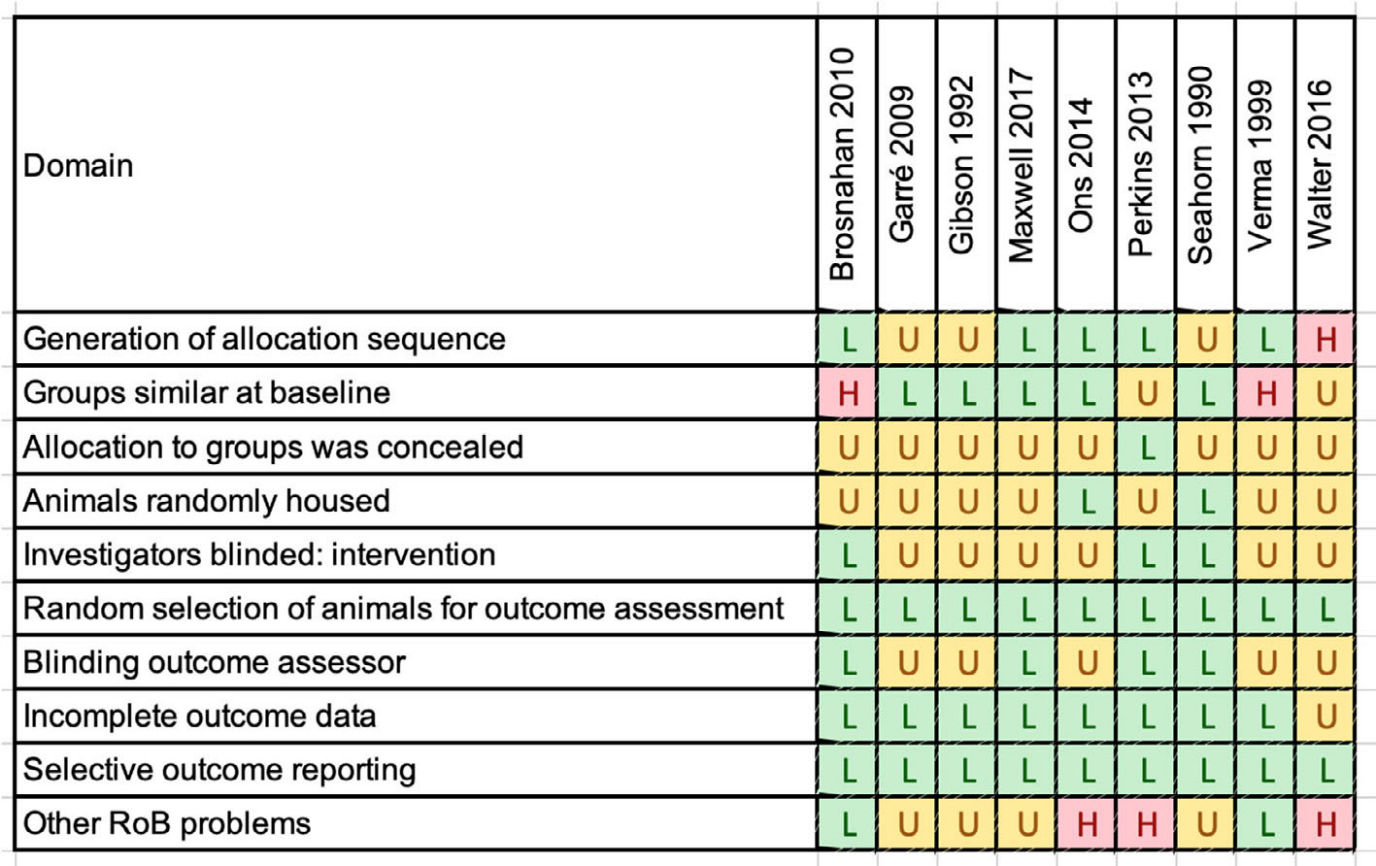
Risk of bias heatmap of included studies. H, high risk of bias; L, low risk of bias; U, unknown risk of bias.

Summary risk‐of‐bias assessments for the included studies are presented in Figure 2. Incomplete reporting of methods in most studies led to an unknown risk of bias for several domains including concealment of animals to experimental groups, random housing of animals, blinding of investigators and outcome assessors, and other problems—most commonly an incomplete description of the possible role of funders. Most studies assessed all animals in the study for all relevant outcomes; thus, incomplete or selective outcome reporting was not identified as a concern in most studies. High risk of bias was noted for several individual domains in 5 studies. Two studies had concerns related to groups being dissimilar at baseline either because of a combination of numerous breeds and ages of animals with unknown vaccination history^25^ or prior exposure history to EHV‐1 in 1 experimental group.^29^ Animals in 1 study were allocated using a non‐random method that depended on their location in a stable resulting in a high risk of bias for this domain.^30^ Concerns about a possible role of the sponsor as well as the presence of concurrent *Streptococcus equi* infection in some study horses were noted for 1 study.^24^ Two horses with ataxia in 1 study were treated with flunixin meglumine raising concern this could affect some clinical outcomes.^25^ Additional confounders, including variable time when heparin was first administered, were also noted as a high risk of bias concern in 1 study.^28^ There were insufficient studies to assess publication bias.

### 3.4 Summary of findings and rating the quality of evidence

Table 1 provides a summary of the main characteristics of the included studies. Table 2 provides a summary of the main findings reported in each of the included studies. Table 3 provides the quality of evidence rating for each of the studies and the 3 main outcomes of interest: clinical signs and pyrexia, signs of neurological disease, and viremia or nasal shedding. Human interferon‐a,^28^ and the cytosine analogue (S)‐1‐[(3‐hydroxy‐2‐phosphonyl methoxy) propyl] cytosine,^16^ failed to elicit significant benefit with respect to reducing either the incidence of fever, signs of neurological disease, or viremia following EHV‐1 exposure. Our confidence in these studies was unaffected by a concern about consistency because of a lack of data from replicate studies. Signs of neurological disease were reported to occur in 1 horse; however, details regarding neurologic exam methods were lacking. Our confidence in the randomized placebo‐controlled study that assessed the efficacy of alpha human interferon^26^ was moderate for all 3 outcomes of interest.

**TABLE 2:** Outcomes assessed and key findings of included studies.

Study	Study comparison	Outcomes assessed	Assessment period	Main findings
Garré et al. (2009)	VAL vs PBO	Pyrexia	0 to 21 dpi	Incidence: Rx: 4/4, PBO 4/4 (OR = 1.0 [0.02 to 62], *P* > .99)
		Rectal temperature		↓ Average rectal temperature at 2 dpi (Rx average = 38.8°C; PBO average = 39.9°C)
		Total fever days		NSD
		Neurologic signs (ataxia or paresis)		Incidence: Rx: 0/4, PBO 0/4 (OR = 1.0 [0.02 to 62], *P* > .99)
		PBMC viremia		Incidence: Rx: 4/4, PBO 4/4 (OR = 1.0 [0.02 to 62], *P* > .99)
		Nasal viral shedding		Incidence: Rx: 4/4, PBO 4/4 (OR = 1.0 [0.02 to 62], *P* > .99)
Maxwell et al. (2017)	VAL (1 week) vs PBO	Rectal temperature	−2 to 14 dpi	NSD
		Mean daily clinical score	2 to 14 dpi	NSD
		PBMC viremia	0 to 14 dpi	Incidence of viremia: Rx: 6/6, PBO 6/6 (OR = 1.0 [0.02 to 58], *P* > .99) ↓ Level of viremia (both groups *P* < .05) at 5 and 6 dpi
		CSF viral titer	15 dpi	NSD
		Nasal viral DNA	−1 to 14 dpi	Incidence of nasal shedding: Rx: 6/6, PBO 6/6 (OR = 1.0 [0.02 to 58], *P* > .99) ↓ Virus in nasal swabs (prophylactic group *P* = .001)
		Neuropathology	15 dpi	Brain lesion incidence: Rx: 0/6, PBO 0/6 (OR = 1.0 [0.02 to 58], *P* > .99); spinal cord lesion incidence: Rx: 1/6, PBO 1/6 (OR = 1.0 [0.05 to 21], *P* > .99)
	VAL (2 week) vs PBO	Rectal temperature	−2 to 14 dpi	↓ Mean rectal temperature (prophylactic group, *P* = .001)
		Mean daily clinical score	2 to 14 dpi	↓ Mean daily clinical score (prophylactic group, *P* = .02)
		PBMC viremia	0 to 14 dpi	Incidence of viremia: Rx: 5/5, PBO 6/6 (OR = 0.85 [0.01 to 50], *P* = .94) ↓ Level of viremia (both groups *P* < .05) at 5 and 6 dpi. ↓ Level of viremia (prophylactic group *P* < .05) at 7 to 10 dpi. ↓ Level of viremia (febrile group *P* < .05) at 10 dpi.
		CSF viral titer	15 dpi	NSD
		Nasal viral DNA	−1 to 14 dpi	Incidence of nasal shedding: Rx: 5/5, PBO 6/6 (OR = 0.85 [0.01 to 50], *P* = .94) ↓ Virus in nasal swabs (prophylactic group *P* = .0008; febrile group *P* = .001)
		Neuropathology	15 dpi	Brain lesion incidence: Rx: 0/5, PBO 0/6 (OR = 1.2 [0.02 to 70], *P* = .94); spinal cord lesion incidence: Rx: 0/5, PBO 1/6 (OR = 0.33 [0.01 to 10], *P* = .53)
	VAL (1 and 2 week) vs PBO	Fever	−2 to 14 dpi	Incidence: Rx (prophylactic group): 4/6, PBO 6/6 (OR = 0.14 [0.005 to 3.63], *P* = .24)
		Ataxia score	−2 to 14 dpi	↓ Ataxia score; *P* = .02
		Euthanasia because of neurologic effects		Incidence: Rx: 0/11, PBO 2/6 (OR = 0.08 [0.003 to 2.0], *P* = .12)
Brosnahan et al. (2010)	Combined sigB3 and siOri2 vs siLuc	Fever and clinical signs	1 to 21 dpi	Incidence: Rx: 10/10, PBO 4/4 (OR = 1.0 [0.019 to 5.15], *P* > .99)
		Neurologic signs	1 to 21 dpi	Incidence: Rx: 2/10, PBO 3/4 (OR = 0.08 [0.005 to 1.294], *P* = .08) Incidence of severe neurologic signs in neurologic horses: Rx: 0/2, PBO 3/3 (OR = 0.03 [0.0004 to 1.99], *P* = .10)
		PBMC viremia	−1 to 21 dpi	Incidence: Rx: 10/10, PBO 4/4 (OR = 2.3 [0.040 to 137], *P* = .68). EHV‐1 in PBMCs by qPCR: NSD
		Nasal shedding	−1 to 21 dpi	Incidence: Rx: 10/10, PBO 4/4 (OR = 2.3 [0.040 to 137], *P* = .68). EHV‐1 in nasal swabs by qPCR: NSD
		Euthanasia because of intractable neurologic disease		Incidence: Rx: 0/10, PBO 3/4 (OR = 0.02 [0.0007 to 0.62], *P* = .03).
Perkins et al. (2013)	Combined sigB3 and siOri2 vs siLuc	Clinical signs or fever	−2 to 21 dpi	Incidence: Rx: 7/7, PBO 6/6 (OR = 1.2 [0.02 to 67], *P* = .95)
		Neurologic signs	−2 to 21 dpi	Incidence: Rx: 3/7, PBO 2/6 (OR = 0.06 [0.002 to 1.46], *P* = .08). No significant (*P* = .88) difference in the severity of the neurologic scores.
		PBMC viremia	−1 to 21 dpi	Incidence: Rx: 7/7, PBO 6/6 (OR = 1.2 [0.02 to 67], *P* = .95). Maximum amount or number of positive samples: NSD
		Nasal viral shedding	−1 to 21 dpi	Incidence: Rx: 7/7, PBO 6/6 (OR = 1.2 [0.02 to 67], *P* = .95). Maximum amount or number of days shedding virus: NSD
		Serum SN titer	−1 to 21 dpi	NSD
Gibson et al. (1992)	HPMPC vs PBO	Clinical signs or fever	1 to 5 dpi	Incidence: Rx: 2/2, PBO 3/3 (OR = 0.71 [0.01 to 50], *P* = .88)
		Nasal viral shedding	−2 to 15 dpi	Incidence: Rx: 2/2, PBO 3/3 (OR = 0.71 [0.01 to 50], *P* = .88)
		WBC viremia	3 to 11 dpi	Incidence: Rx: 2/2, PBO 2/3 (OR = 3.0 [0.08 to 115], *P* = .56)
Ons et al. (2014)	iPPVO vs PBO	Fever	−2 to 28 dpi	Incidence on 1 dpi: Rx: 2/11, PBO 6/12 (OR = 0.22 [0.03 to 1.49], *P* = .12)
		Rectal temperature and clinical signs		Transient changes. Mean rectal temperature ↓ at 11‐13 dpi (*P* = .02 to .04). ↓ Incidence nasal discharge at 11 dpi (*P* = .03). ↓ Incidence of lymphadenopathy at 11, 17, and 19 dpi; (*P* = .04 to .05)
		Nasal viral shedding	0 to 21 dpi	Incidence: Rx: 7/11, PBO 6/12 (OR = 1.75 [0.33 to 9.3], *P* = .51). ↓ Virus detection at 11–16 dpi (*P* = .002 to .013)
Seahorn et al. (1990)	α‐2a IFN vs PBO (pooled data)	Fever	−4 to 14 dpi	Incidence: Rx: 10/12, PBO 5/6 (OR = 1.0 [0.07 to 13.9], *P* > .99)
		Neurologic signs	2 to 14 dpi	Incidence: Rx: 0/12, PBO 1/6 (OR = 0.15 [0.005 to 4.20], *P* = .26)
		PBMC viremia	2 to 14 dpi	Incidence: Rx: 5/12, PBO 3/6 (OR = 0.71 [0.10 to 5.12], *P* = .74)
		Nasal viral shedding	−2 to 10 dpi	Incidence: Rx: 12/12, PBO 6/6 (OR = 1.92 [0.034 to 109], *P* = .75)
Verma et al. (1999)	Herbal product vs PBO	Fever or clinical signs	0 to 21 dpi	Incidence: Rx: 0/2, PBO 2/2 (OR = 0.04 [0.0005 to 2.93], *P* = .14)
		WBC viremia		Incidence: Rx: 0/2, PBO 2/2 (OR = 0.04 [0.0005 to 2.93], *P* = .14)
		Nasal shedding		Incidence: Rx: 1/2, PBO 2/2 (OR = 0.20 [0.005 to 8.83], *P* = .40)
Walter et al. (2016)	Heparin vs no treatment	Incidence of EHM, Days 1‐28 of the febrile outbreak, ↓ incidence, heparin, *P* = .03	Days 1‐28 of the febrile outbreak	Incidence: Rx: 1/31, PBO 7/30 (OR = 0.11 [0.013 to 0.954], *P* = .05).

Abbreviations: d, day; dpi, day post infection; EHM, equine herpesvirus myeloencephalopathy; gB3, glycoprotein B; HPMPC, (S)‐l‐[(3‐hydroxy‐2‐phosphonyl methoxy) propyl] cytosine; IFN, interferon; iPPVO, inactivated *Parapoxvirus ovis* (Zylexis^â^); Luc, firefly luciferase; NR, not reported; Ori2, origin binding protein helicase; PBO, placebo (control); Rx, treatment; si, small interfering; siRNA, small interfering RNA; VAL, valacyclovir.

**TABLE 3:** Summary of evidence for the efficacy of prophylactic use of therapeutics for EHV‐1 infection in horses.

Treatment	Studies	Type of evidence and initial rating based on presence of key features	Outcome: Reduction in	Incidence OR^a^	Quality	Consistency	Directness	Precision	Final score and rating	Comments
VAL	(2) Garré et al. (2009) Maxwell et al. (2017)	Randomized placebo‐controlled experimental study (start = ++++)	Clinical signs or pyrexia	0.14 to 1.0	↓	↓	0	↓	Very low	Moderate risk of bias (blinding). Unexplained inconsistent results. Negative results (small sample sizes).
			Neurologic signs	1.0	↓	0	0	↓	Low	Moderate risk of bias (blinding). Negative results (small sample sizes).
			Viremia	1.0	0	0	0	↓	Moderate	Negative results for incidence (small sample sizes). One study showed decreased viremia levels.
siRNA for gB3 and iOri2	(2) Brosnahan et al. (2010) Perkins et al. (2013)	Randomized, placebo‐controlled experimental studies (start = ++++)	Clinical signs or pyrexia	1.0 to 1.2	0	0	0	↓	Moderate	Negative results (small sample sizes).
			Neurologic signs	0.06 to 0.08	0	0	0	↓	Moderate	Unexplained inconsistent results. Negative results for incidence (small sample sizes). One study showed a decrease in incidence because of intractable neurologic disease.
			Viremia	1.2 to 2.3	0	0	0	↓	Moderate	Negative results for incidence (small sample sizes).
α‐2a IFN	(1) Seahorn et al. (2010)	Randomized, blinded, placebo‐controlled experimental study (start = ++++)	Pyrexia	1.0	0	0	0	↓	Moderate	Negative results for incidence (small sample sizes).
			Neurologic signs	0.15	0	0	0	↓	Moderate	Negative results for incidence (small sample sizes).
			Viremia	0.71	0	0	0	↓	Moderate	Negative results for incidence (small sample sizes).
iPPVO	(1) Ons et al. (2010)	Randomized, placebo‐controlled experimental study (start = ++++)	Clinical signs or pyrexia	0.71	↓↓	0	0	↓	Very low	High risk of bias (blinding; other problem notably *S. equi* coinfection). Negative results for incidence (small sample sizes).
			Nasal shedding	1.75	0	0	0	↓	Moderate	Negative results for incidence (small sample sizes).
HPMPC	(1) Gibson et al. (1992)	Placebo‐controlled experimental study (start = +++)	Clinical signs or pyrexia	0.71	↓	0	0	↓	Very low	Moderate risk of bias (blinding). Negative results for incidence (small sample sizes).
			Viremia	3.0	0	0	0	↓	Low	Negative results for incidence (small sample sizes).
Herbal product	(1) Verma et al. (1999)	Placebo‐controlled (start = +++)	Clinical signs or pyrexia	0.04	↓↓	0	0	↓	Very low	High risk of bias (blinding, groups similar at baseline). Negative results for incidence (small sample sizes). Upgrade (↑) for magnitude of effect.
			Viremia	0.04	↓	0	0	↓	Very low	Moderate risk of bias (groups similar at baseline). Negative results for incidence (small sample sizes).

Abbreviations: gB3, glycoprotein B; HPMPC, (S)‐l‐[(3‐hydroxy‐2‐phosphonyl methoxy) propyl] cytosine; IFN, interferon; iPPVO, inactivated *Parapoxvirus ovis* (Zylexis^â^); OR, odds ratio; Ori2, origin binding protein helicase; siRNA, small interfering RNA; VAL, valacyclovir.

^a^ See Table 2 for confidence intervals.

A plant extract derived in part from *Withanis somnifera*, *Ocimum sanctum*, *Emblica officinale*, and *Tinospora cardiofolia*,^29^ also failed to demonstrate a significant benefit with respect to reducing either the incidence or severity of signs of respiratory disease or fever or viremia following EHV‐1 exposure. This negative study was downgraded for concerns related to precision because of small sample sizes and insufficient power. This study was downgraded up to twice for study quality concerns related to risk of bias related to blinding and similarity of the groups at baseline.^29^ Downgrades arising from concerns about blinding were applied for the clinical outcomes rather than assessment of viremia or nasal shedding of virus where blinding was less likely to have an impact. Our confidence in the placebo‐controlled study that assessed the efficacy of the plant extract^29^ was very low for signs of respiratory disease and pyrexia and low for viremia.

Two studies investigated the efficacy of prophylactic intranasal administration of small inhibitory RNAs^25^, ^27^ that targeted genes encoding glycoprotein B (*gB3*) and the origin binding protein helicase (*Ori2*). These genes are required for replication of the EHV‐1 genome (Ori) or virus entry and cell‐to‐cell transmission (gB), respectively.^25^ In 1 randomized blinded placebo‐controlled study, 750 pmoles of each small inhibitory RNA were given 24 hours apart starting at 12 hours before EHV‐1 exposure.^25^ This treatment did not affect the duration of viremia or nasal shedding, CSF cytology, number of fever days, or the incidence of clinical signs including signs of neurological disease. The treatment did, however, decrease the number of animals that required euthanasia because of severe neurological disease.^25^ A subsequent randomized placebo‐controlled study^27^ using the same small inhibitory RNAs at a lower dose (20‐30 pmoles) given 5 times (every 12 hours starting at 1 day before EHV‐1 infection) failed to demonstrate any beneficial clinical effects or changes in either duration of viremia or nasal shedding of the virus. Our confidence in these studies was decreased because of concerns about risk of bias attributed to a lack of blinding, and unexplained inconsistent result that was often expressed as significant changes occurring on a single study day, and small sample sizes that contributed to a lack of precision in the estimate of the effect. The net effect was that our confidence in these studies were very low for both signs of respiratory disease and pyrexia and neurologic effects and low for any therapeutic benefit for either viremia or nasal shedding of the EHV‐1 virus.

One randomized placebo‐controlled study^26^ investigated a commercially available immunomodulator containing inactivated *Parapoxvirus ovis* (Zylexis). This study administered the immunomodulator (2 mL, intramuscular) starting at 2 days before EHV‐1 infection. Administration of the immunomodulator was repeated on the day of infection and at 7 days post‐infection. The treatment decreased the amount of virus shedding in nasal mucus, reduced fever days, and also reduced the incidence of lymphadenopathy and nasal discharge seen on 1 or more days after EHV‐1 infection. Horses on this study developed *Streptococcus equi equi* infections on Day 3 after EHV‐1 infection confounding the analysis of some of the clinical data. Our confidence in this placebo‐controlled study was decreased because of concerns about risk of bias attributed to both a lack of blinding and confounding effects of *S. equi* infection, and small sample sizes that contributed to a lack of precision in the effect estimate. The net effect was that our confidence in this study was very low for signs of respiratory disease and pyrexia and moderate for a therapeutic benefit for viremia or nasal shedding of the EHV‐1 virus. This study did not assess neurologic outcomes.

Two studies investigated whether oral valacyclovir was beneficial in the control of EHV‐1 infection outcomes. In 1 placebo‐controlled study,^12^ horses were given 40 mg/kg valacyclovir 3 times daily for either 5 or 7 days. In this study, valacyclovir was first administered 1 hour before EHV‐1 infection. Except for a short‐term reduction in rectal temperature seen on Day 2 post‐infection, horses given 40 mg/kg valacyclovir did not demonstrate either fewer days of viremia, reduced nasal shedding, or other clinical benefits.^12^ A subsequent blinded randomized placebo‐controlled study examined the benefit of valacyclovir when given either prophylactically (starting at 1 day before EHV‐1 infection) or to febrile horses after EHV‐1 infection.^14^ Horses in either experimental arm were given a loading dose of 27 mg/kg, 3 times a day, for 2 days. Maintenance doses used in this study were 18 mg/kg and these were given every 12 hours for either 1 or 2 weeks. Valacyclovir treatment, when given either prophylactically or to febrile horses, reduced viremia and nasal shedding of the virus when compared with placebo‐treated horses. Prophylactic administration of valacyclovir for 2 weeks also reduced rectal temperature in infected horses and reduced signs of respiratory disease. Analysis of the combined 1‐ and 2‐week data from the prophylactic trial also showed that valacyclovir reduced the incidence of ataxia in infected horses.^14^ Administration of valacyclovir to febrile horses had no effect on signs of respiratory or neurological disease, or fever. Our confidence in these studies was decreased because of moderate concerns about risk of bias attributed to a lack of blinding, and small sample sizes that contributed to a lack of precision in the estimate of the effect. The studies were also downgraded for unexplained inconsistencies of the results for pyrexia. Our confidence in these studies for the main outcomes was very low for fever and low for signs of neurological disease. These studies also reported therapeutic benefits for either viremia or nasal shedding of the EHV‐1 virus, and the small sample sizes raised concerns about precision. When applied, the downgrades reduced our level of confidence in these studies was moderate for benefits associated with valacyclovir and viremia and nasal shedding.

One observational study^28^ evaluated the efficacy of subcutaneous heparin (25 000 IU, 2 times a day for 3 days) during a naturally occurring outbreak with a neuropathogenic G2254/D752 Pol variant of EHV‐1. In this study, febrile horses were either treated with heparin or were untreated. Treatment outcomes were analyzed retrospectively and assignment to treatment groups was not randomized. When compared with untreated horses, horses given heparin had a lower incidence (3.2%) of EHM when compared with untreated horses (23.3%; *P* = .03). As an observational study, our initial confidence in this study was given a rating of 2. Multiple downgrades (−2) occurred because of a high degree of concern for risk of bias arising from inadequate randomization, lack of blinding, separate housing for controls, and movement between groups. Since this study has not been replicated no downgrades were applied for consistency. Likewise, downgrades for precision or directness were not applied. The confidence rating was upgraded 1 grade (+1) for a large magnitude of effect yielding a final confidence rating of 1 (very low) for a benefit associated with heparin treatment and the incidence of EHM in horses with naturally occurring EHV‐1 infection.

## 4 DISCUSSION

This systematic review provides the result of our search of electronic and print resources with peer‐reviewed publications in any language and without restriction to publication date. One limitation of our systematic review is that we did not include conference proceedings, technical reports, and other gray literature in the review, although some studies have shown that reports published solely in the gray literature can have methodological weaknesses and high risk of bias.^31^ This review yielded a small number of studies investigating therapeutic benefits of 7 different drug classes. Replicate studies were unavailable for 5 of these drug classes addressed in our review. Because the drugs included in this review have different pharmacologic modes of action, we were unable to pool the data across different drug classes in a clinically meaningful way. This review did address vaccinations as a therapeutic modality; these were the subject of systematic review recently published by the study authors in this journal, and others.^32^, ^33^

We found that reporting of the methodological features of studies included in this systematic review was often incomplete, making evaluation of risk of bias within and across studies difficult. Because of this, it is possible that studies received a lower individual grading for bias than existed. Study authors were not contacted to verify aspects of study design not clearly reported in the published article. Incomplete reporting of methodological details in experimental animal studies and animal‐centric systematic reviews have been noted by others.^33^, ^34^, ^35^, ^36^, ^37^ Increased adherence of study authors to suggested reporting guidelines^38^ remains urgent in the veterinary literature. Another important limitation of our study is that multiple breeds of horses of different age groups were also used reducing our ability to generalize our findings to the general horse population that could be infected with EHV‐1.

Since the conduct of the literature searches underlying our study 1 notable publication has appeared which evaluated oral administration of valganciclovir in a randomized clinical trial to measure the effect on clinical signs of disease, viral infection and seroconversion when administered immediately after experimental EHV‐1 infection.^39^ Eight ponies were randomly assigned to either treatment or a control group; only the laboratory component of the study was blinded.

One strength of the included studies was the presence of treatment and control groups. However, this strength was offset by the observation that many of the included studies had presumed low statistical power because of a possible combination of small sample size and small effect size. Indeed, multiple studies^12^, ^14^, ^16^, ^25^, ^29^ had treatment and placebo group sizes with fewer than 5 horses in either a treatment or placebo group which is likely to seriously limit the detection of any treatment benefit. This is likely an outcome of the costs of conducting therapeutic studies in horses. This is compounded by the low incidence of EHM in experimental trials reducing the ability of studies to detect a treatment effect. Therefore, experimental studies evaluating whether a treatment will reduce the incidence of EHM can require relatively large sample sizes. The net effect of insufficient sample sizes was that serious imprecision affected studies included in this review. This deficit has also been noted in intervention studies performed in other veterinary species.^40^

The limitations noted in our review reduced our confidence in the reviewed studies. For most of the main outcomes our confidence in the studies were rated as low to very low, especially with respect to EHM and other disease outcomes. Very few drug classes had studies which replicated results. In the 2 cases where replicate studies were available for either valacyclovir or the use of an siRNA for gB3 and iOri2, these studies varied with respect to animal populations, drug dosages, and certain outcome measures. This introduces some inherent heterogeneity in study results. Our findings strongly suggest that there is an ongoing need for additional randomized and blinded studies for the use of the drugs evaluated in this review of EHV‐1 infection.

## CONFLICT OF INTEREST DECLARATION

Authors declare no conflict of interest.

## OFF‐LABEL ANTIMICROBIAL DECLARATION

Authors declare no off‐label use of antimicrobials.

## INSTITUTIONAL ANIMAL CARE AND USE COMMITTEE (IACUC) OR OTHER APPROVAL DECLARATION

Authors declare no IACUC or other approval was needed.

## HUMAN ETHICS APPROVAL DECLARATION

Authors declare human ethics approval was not needed for this study.

## Supporting information


**Supplementary Item 1:** Study protocol.
**Supplementary Item 2:** Search strategy.
**Supplementary Item 3:** List of studies that were excluded based on a review of the full text. The reason for exclusion is also provided.
**Supplementary Item 4:** List of included studies and funding source.
**Supplementary Item 5:** Demographics and pre‐study EHV‐1 status of included studies.
