# Supplementary material for: Pharmacologic interventions for the treatment of equine herpesvirus‐1 in domesticated horses: A systematic review
Source: J Vet Intern Med. 2024 Feb 21;38(3):1892–905. doi: 10.1111/jvim.17016 (PMC11099759; doi:10.1111/jvim.17016)
Supplement: Supplementary file 1 — Supplementary Item 1: Study protocol. Supplementary Item 2: Search strategy. Supplementary Item 3: List of studies that were excluded based on a review of the full text. The reason for exclusion is also provided. Supplementary Item 4: List of included studies and funding source. Supplementary Item 5: Demographics and pre‐study EHV‐1 status of included studies. [file JVIM-38-1892-s001.pdf]

Supplementary Item 1: Study Protocol

Title: Pharmacologic interventions for the management of equine herpesvirus-1 in domesticated horses: A systematic review

Research question: Does pharmacological therapy with antivirals, NSAIDs, corticosteroids, anti-coagulants, or other therapies decrease either the incidence or severity of disease and infection of EHV-1 in domesticated horses?

Problem formulation: The review question was developed and refined through a series of problem formulation steps including preliminary literature searches.

Searches: The review team will consider using existing systematic reviews to address or help to address its research question. English-language systematic reviews conducted within the last 5 years will be sought using searches in PubMed, PROSPERO (CRD), and CAMARADES. A search for bibliographic references will be performed with the assistance of an experienced librarian through MEDLINE (via PubMed), LILACS (via Virtual Health Library), Cochrane Library (via Virtual Health Library) and EMBASE to locate randomized controlled trials and other studies. The search will be limited to domesticated horses and performed without sex, age, breed, or language restrictions. Only peer-reviewed publications will be considered. The search strategies will include the search for descriptors or words in the text related to the disease and the type of intervention. The search will be developed with input from a librarian with expertise in the conduct of systematic reviews.

Types of studies to be included: Randomized controlled trials and observational studies.

Condition or domain being studied: Equine herpesvirus-1 (EHV-1) is a highly prevalent Alphaherpesviridae virus that infects horses worldwide. This respiratory virus is transmitted via direct horse-to-horse contact with contaminated nasal secretion as well as indirectly from contact with contaminated aborted fetuses, placenta, and fomites. This virus is associated with equine herpesvirus-1

myeloencephalopathy (EHM), respiratory disease, abortion, and neonatal death among other outcomes. Treatment is primarily supportive, and usually includes anti-inflammatories and fluids to maintain hydration. This review considers other drug therapies for the management of EHV-1 infection in horses.

PICO Statement

- **Population**: Domesticated equids without sex, age, or breed restrictions
- **Intervention:** Any drug therapy, irrespective of dose, route of administration, or duration of Tx, given at any of the following points:
  - 1. Prophylactic treatment in advance of EHV-1 infection
  - 2. Treatment post-infection EHV-1, in the absence of neurological signs
  - 3. Treatment post-infection EHV-1, in the presence of neurological signs (EHM: ataxia and weakness)
- **Comparator:** Horses infected or exposed to EHV-1 infection, and treated with any drug treatment, compared to placebo-treated horses, or other dosage of the same treatment (dose response).
- **Outcome:** All clinical outcomes that reflect symptomatic EHV-1 infection or viral infection. Presence and degree of viral infection.

Inclusion and Exclusion Criteria

- **Inclusion criteria:**
  - Domesticated equids without sex, age, breed, or immunological status restriction
  - Therapeutic trials that evaluated the efficacy of a drug against EHV-1 experimental or natural infection
  - Studies that used a placebo or other dosage of same drug.
  - Study included clinical outcomes that reflect symptomatic EHV-1 infection.
  - Endpoints related to drug efficacy: relative reduction in EHV-1 disease risk; reduction in odds of EHV-1 infection
  - Studies will not be excluded on the basis of year, language, or quality

- **Exclusion criteria:**
  - Absence of an EHV-1 challenge trial of drug efficacy
  - Absence of the selected clinical or virological outcomes
  - Wrong species of virus
  - Lack of concurrent control or comparator
  - Wrong species (not equid)
  - Purely descriptive observational studies
  - No original data

Reason for exclusion for all studies will be recorded.

Main outcomes:

- Rhinopneumonitis: pyrexia with respiratory signs, including oculo-nasal discharge, elevated respiratory rate, cough, lethargy
- Abortion in the third trimester
- Equine Herpes Myeloencephalopathy
- Neonatal infection
- Ocular disease
- Male reproductive tract infection - orchitis

Review team: The review team will include Peggy Gross (Librarian), David Dorman, Paul Lunn, and Lutz Goehring. If a member of the review team was a coauthor of a study under review, that member will recuse himself or herself from the evaluation of the quality of that study. The review team will be responsible for performing all aspects of the review, including conducting the literature searches; applying inclusion/exclusion criteria to screen studies; extracting data; assessing risk of bias for included studies; and analyzing and synthesizing data. The roles and responsibilities of the team members will be documented throughout the protocol. Throughout the course of its work, the review team will also engage others as needed. The involvement of those individuals will be documented.

Study selection

The evaluation of titles, abstracts, and the full text will be independently performed by two reviewers (DD, PL, LG); disagreements will be resolved by a third reviewer. A database management system (Covidence) will be used to manage and document these decisions.

Data extraction: Data will be collected and recorded (i.e., extracted) from included studies by one member of the review team (DCD, to be determined) and checked by a second member for completeness and accuracy (to be determined). Any discrepancies in data extraction will be resolved through discussion. The extracted data will be used to summarize study designs and findings and/or to conduct statistical analyses.

Specific study endpoints to be extracted include:

Treatment protocol:

- Drug
- Dosage protocol
- Timing of drug administration relative to EHV-1 infection or EHM occurrence

Challenge protocol:

- Virus challenge used
- Virus dose and route of administration

Clinical signs (Yes/No, duration in days, severity):

- Fever (rectal temperature >38.6°C)
- Clinical score – based on some combination of nasal discharge, cough, tachypnea/dyspnea, anorexia, lethargy
- Neurologic disease, for example: ataxia; weakness; and urinary incontinence

Clinical Pathology

- Leukogram changes or blood changes indicative of inflammatory disease
- Cerebrospinal fluid color, cytology, and protein concentration

Pathology

- Nervous system pathology

- Upper respiratory tract pathology
- Reproductive tract pathology
- Airway pathology
- Ocular pathology

Virology (Yes/No, duration in days, quantitation):

- Viral culture from nasal/naso-pharyngeal swabs and methods used
- Viral DNA in nasal swab samples and method used
- Viral culture from blood and methods used
- Viral DNA in blood and method used
- Viral DNA in tissues
- IHC in Tissues

Risk of bias evaluation: The risk of bias domains and questions are based on established guidance for animal studies. [1] The SYRCLE risk of bias tool includes a common set of questions that are answered based on the specific details of individual studies to develop risk of bias ratings (using the following three options: low risk of bias; unknown risk of bias; or high risk of bias). Information or study procedures that were not reported are assumed not to have been conducted, resulting in an assessment of "unknown" risk of bias. Studies will be independently assessed by two assessors (to be determined) who answer all applicable risk of bias questions with one of three options following prespecified criteria. Risk of bias will be assessed at the outcome level. After assessors have independently made risk of bias determinations for a study across all risk of bias questions, the two assessors will compare their results to identify discrepancies and attempt to resolve them. Any remaining discrepancies will be considered and resolved with the review team. The final risk of bias rating for each question will be recorded along with a statement of the basis for that rating. All risk of bias assessments will be recorded using Covidence. The following domains will be assessed:

- Random sequence generation
- Allocation sequence
- Blinding of participants and personnel

- Blinding of outcome assessment
- Incomplete outcome data
- Selective reporting
- Other bias

Strategy of data synthesis: A narrative synthesis (e.g., study design, year of publication, subject baseline demographics, sample size, country where study was conducted, interventions, and the results from each study) will be performed for each intervention. If we identify a sufficient number of studies with adequate homogeneity, a meta-analysis will also be considered.

Confidence rating: Assessment of the body of evidence: The quality of evidence for each therapy will be evaluated using the GRADE system for rating the confidence in the body of evidence. [2,3] In brief, available studies on a particular outcome and drug will be initially grouped by key study-design features, and each grouping of studies is given an initial confidence rating by those features. The initial rating is downgraded for factors that decrease confidence in the results, including risk of bias, unexplained inconsistency, indirectness or lack of applicability, imprecision, or publication bias. The initial rating is upgraded for factors that increase confidence in the results, including large magnitude of effect, dose response, consistency across study designs/populations/animal models or species, consideration of residual confounding, other factors that increase confidence in the association or effect (e.g., particularly rare outcomes). Confidence ratings are independently assessed by members of the review team, and discrepancies will be resolved by consensus as needed. Confidence ratings will be summarized in evidence profile tables.

References:

1. Hooijmans CR, Rovers MM, de Vries RB, et al. SYRCLE's risk of bias tool for animal studies. BMC Med Res Methodol 2014;14:43.

2. Guyatt GH, Oxman AD, Schunemann HJ, et al. GRADE guidelines: a new series of articles in the Journal of Clinical Epidemiology. J Clin Epidemiol 2011;64:380-382.

3. Guyatt GH, Oxman AD, Vist GE, et al. GRADE: an emerging consensus on rating quality of evidence and strength of recommendations. BMJ 2008;336:924-926.

Protocol developed: 23 DEC 19

Protocol Amendments

Risk of bias:

Training manual developed: 19 JUN 2020

Review team:

The following individuals were added to the review team:

- Kelsie Dougherty 28 JUL 2020
  - Roles: Data extraction, risk of bias assessment
- Claire Neinast 4 AUG 2020
  - Roles: Data extraction, risk of bias assessment

Exclusion criteria:

- Only peer-reviewed publications will be considered. Added by working group consensus: 20 AUG 2020

Supplementary Item 2: Search Strategy

PubMed

Concept 1: Pharmacological therapy

"Pharmacologic Actions"[Mesh] OR "pharmacology" [Subheading] OR pharmacological[tw] OR pharmacologic[tw] OR "Antiviral Agents"[Mesh] OR "Antiviral Agents"[tw] OR "Antiviral"[tw] OR "Anti-Infective Agents"[Mesh] OR "Anti-Infective"[tw] OR "Anti-Inflammatory Agents, Non-Steroidal"[Mesh] OR NSAIDs[tw] OR NSAID[tw] OR "Anti-Inflammatory Agents"[Mesh] OR "Anti Inflammatory"[tw] OR "AntiInflammatory"[tw] OR "Antiinflammatories"[tw] OR "Anti inflammatories"[tw] OR "NonSteroidal"[tw] OR "Non Steroidal"[tw] OR corticosteroid[tw] OR corticosteroid[tw] OR corticosteroids[tw] OR cortisone[tw] OR corticoid[tw] OR corticoids[tw] OR "Dexamethasone"[Mesh] OR dexamethasone[tw] OR Methylfluorprednisolone[tw] OR Hexadecadrol[tw] OR Decameth[tw] OR Decaspray[tw] OR Dexasone[tw] OR Dexpak[tw] OR Maxidex[tw] OR Millicorten[tw] OR Oradexon[tw] OR Decaject[tw] OR Hexadrol[tw] OR "Betamethasone"[Mesh] OR Flubenisolone[tw] OR Betadexamethasone[tw] OR Celestona[tw] OR Celeston[tw] OR Celestone[tw] OR betamethasone[tw] OR "Flumethasone"[Mesh] OR flumethasone[tw] OR isoflupredone[tw] OR fludrocortisone[tw] OR "Triamcinolone"[Mesh] OR triamcinolone[tw] OR Volon[tw] OR Aristocort[tw] OR "Prednisolone"[Mesh] OR prednisolone[tw] OR Predate[tw] OR Predonine[tw] OR "Di Adreson F"[tw] OR betamethasone[tw] OR hydrocortisone[tw] OR "Glucocorticoids" [Pharmacological Action] OR "Glucocorticoids"[tw] OR "Glucocorticoid"[tw] OR "Anticoagulants" [Pharmacological Action] OR "Anticoagulants"[Mesh] OR "Anticoagulants"[tw] OR "Anti coagulants"[tw] OR "Anticoagulant"[tw] OR "Anti coagulant"[tw] OR Anticoagulation[tw] OR Anti coagulation[tw] OR "Indirect Thrombin"[tw] OR "Antithrombins"[Mesh] OR "Anti thrombins"[tw] OR "Antithrombins"[tw] OR drugs[tw] OR drug[tw]
not found in PubMed: Cellestoderm[tw], Fluorodexamethasone[tw], AdresonF[tw]

Concept 2:EHV1

"Herpesvirus 1, Equid"[Mesh] OR "equine herpesvirus 1"[tw] OR "equine herpes virus 1"[tw] OR "Equine abortion Virus"[tw] OR "Equine abortion Viruses"[tw] OR "EHV 1"[tw] OR EHV1[tw] OR "equid herpesvirus 1"[tw] OR "equid herpesvirus type 1"[tw] OR "Equine herpesvirus myeloencephalopathy"[tw] OR "Equine herpes myeloencephalopathy"[tw] OR EHM[tw] OR "equine herpesvirus type 1"[tw] OR "equine herpes virus type 1"[tw] OR "alphaherpesvirus"[tw]

Concept 3: Horses

horses[mesh] OR horse[tw] OR horses[tw] OR equid*[tw] OR equine*[tw] OR equus[tw]

Web of Science =451

Limit to: Article, proceedings, review, epub

Concept 1: Pharmacological therapy

TS=("pharmacology" OR pharmacological OR pharmacologic OR "Antiviral" OR "Anti-Infective" OR NSAIDs OR NSAID OR "Anti Inflammatory” OR "AntiInflammatory” OR "Antiinflammatories" OR "Anti inflammatories" OR "NonSteroidal" OR "Non Steroidal" OR corticosteroid OR corticosteroid OR corticosteroids OR cortisone OR corticoid OR corticoids OR dexamethasone OR Methylfluorprednisolone OR Hexadecadrol OR Decameth OR Decaspray OR Dexasone OR Dexpak OR Maxidex OR Millicorten OR Oradexon OR Decaject OR Hexadrol OR Flubenisolone OR Betadexamethasone OR Celestona OR Celeston OR Celestone OR betamethasone OR flumethasone OR isoflupredone OR fludrocortisone OR triamcinolone OR Volon OR Aristocort OR prednisolone OR Predate OR Predonine OR "Di Adreson F” OR betamethasone OR hydrocortisone OR "Glucocorticoids" OR "Glucocorticoid" OR "Anticoagulants" OR "Anti coagulants" OR "Anticoagulant" OR "Anti coagulant" OR Anticoagulation OR Anti coagulation OR “Indirect Thrombin” OR "Anti thrombins" OR "Antithrombins" OR drugs OR drug)

Concept 2:EHV1

TS=(“equine herpesvirus 1” OR “equine herpes virus 1” OR “Equine abortion Virus” OR “Equine abortion Viruses” OR “EHV 1” OR EHV1 OR “equid herpesvirus 1” OR "equid herpesvirus type 1” OR "Equine herpesvirus myeloencephalopathy” OR “Equine herpes myeloencephalopathy” OR EHM OR "equine herpesvirus type 1” OR "equine herpes virus type 1” OR "equid alphaherpesvirus 1” OR “Equine alphaherpesvirus 1”)

Concept 3: Horses

TS=(horse OR horses OR equid* OR equine* OR equus)

Concept 4: article type

NOT

comment[pt] OR editorial[pt] OR letter[pt] OR news[pt] OR “newspaper article”[pt] OR review[pt] OR “systematic review”[pt] OR “meta-analysis”[pt] OR “consensus development conference”[pt] OR guideline[pt] OR “practice guideline”[pt] OR bibliography[pt]

AGRICOLA =35

Concept 1: Pharmacological therapy

"pharmacology" OR pharmacological OR pharmacologic OR "Antiviral" OR "Anti-Infective" OR NSAIDs OR NSAID OR "Anti Inflammatory" OR "AntiInflammatory" OR "Antiinflammatories" OR "Anti inflammatories" OR "NonSteroidal" OR "Non Steroidal" OR corticosteroid OR corticosteroid OR corticosteroids OR cortisone OR corticoid OR corticoids OR dexamethasone OR Methylfluorprednisolone OR Hexadecadrol OR Decameth OR Decaspray OR Dexasone OR Dexpak OR Maxidex OR Millicorten OR Oradexon OR Decaject OR Hexadrol OR Flubenisolone OR Betadexamethasone OR Celestona OR Celeston OR Celestone OR betamethasone OR flumethasone OR isoflupredone OR fludrocortisone OR triamcinolone OR Volon OR Aristocort OR prednisolone OR Predate OR Predonine OR "Di Adreson F" OR betamethasone OR hydrocortisone OR "Glucocorticoids" OR "Glucocorticoid" OR "Anticoagulants" OR "Anti coagulants" OR "Anticoagulant" OR "Anti coagulant" OR Anticoagulation OR Anti coagulation OR "Indirect Thrombin" OR "Anti thrombins" OR "Antithrombins" OR drugs OR drug

Concept 2:EHV1

"equine herpesvirus 1" OR "equine herpes virus 1" OR "Equine abortion Virus" OR "Equine abortion Viruses" OR "EHV 1" OR EHV1 OR "equid herpesvirus 1" OR "equid herpesvirus type 1" OR "Equine herpesvirus myeloencephalopathy" OR "Equine herpes myeloencephalopathy" OR EHM OR "equine herpesvirus type 1" OR "equine herpes virus type 1" OR "equid alphaherpesvirus 1" OR "Equine alphaherpesvirus 1"

Concept 3: Horses

horse OR horses OR equid* OR equine* OR equus

Concept 4: article type

NOT

comment[pt] OR editorial[pt] OR letter[pt] OR news[pt] OR "newspaper article"[pt] OR review[pt] OR "systematic review"[pt] OR "meta-analysis"[pt] OR "consensus development conference"[pt] OR guideline[pt] OR "practice guideline"[pt] OR bibliography[pt]

CAB Abstracts = 259

Concept 1: Pharmacological therapy

"pharmacology" OR "pharmacological" OR "pharmacologic" OR "Antiviral" OR "Anti-Infective" OR "NSAIDs" OR "NSAID" OR "Anti Inflammatory" OR "AntiInflammatory" OR "Antiinflammatories" OR "Anti inflammatories" OR "NonSteroidal" OR "Non Steroidal" OR "corticosteroid" OR "corticosteroid" OR "corticosteroids" OR "cortisone" OR "corticoid" OR "corticoids" OR "dexamethasone" OR "Methylfluorprednisolone" OR "Hexadecadrol" OR "Decameth" OR "Decaspray" OR "Dexasone" OR "Dexpak" OR "Maxidex" OR "Millicorten" OR "Oradexon" OR "Decaject" OR "Hexadrol" OR "Flubenisolone" OR "Betadexamethasone" OR "Celestona" OR "Celeston" OR "Celestone" OR "betamethasone" OR "flumethasone" OR "isoflupredone" OR "fludrocortisone" OR "triamcinolone" OR "Volon" OR "Aristocort" OR "prednisolone" OR "Predate" OR "Predonine" OR "Di Adreson F" OR "betamethasone" OR hydrocortisone OR "Glucocorticoids" OR "Glucocorticoid" OR "Anticoagulants" OR "Anti coagulants" OR "Anticoagulant" OR "Anti coagulant" OR "Anticoagulation" OR "Anti coagulation" OR "Indirect Thrombin" OR "Anti thrombins" OR "Antithrombins" OR "drugs" OR "drug"

Concept 2:EHV1
“equine herpesvirus 1” OR “equine herpes virus 1” OR “Equine abortion Virus” OR “Equine abortion Viruses” OR “EHV 1” OR EHV1 OR “equid herpesvirus 1” OR "equid herpesvirus type 1” OR "Equine herpesvirus myeloencephalopathy” OR “Equine herpes myeloencephalopathy” OR EHM OR "equine herpesvirus type 1” OR "equine herpes virus type 1” OR "equid alphaherpesvirus 1” OR “Equine alphaherpesvirus 1”

Concept 3: Horses
horse OR horses OR equid* OR equine* OR equus

Global Index Medicus regional databases:
4 citations
African Index Medicus (AIM), Eastern Mediterranean Region (IMEMR), South-East Asia Region (IMSEAR), Latin America and the Caribbean Literature on Health Sciences (LILACS), Western Pacific Region Index Medicus (WPRO)
"pharmacology" OR "pharmacological" OR "pharmacologic" OR "Antiviral" OR "Anti Infective" OR "NSAIDs" OR "NSAID" OR "Anti Inflammatory" OR "AntiInflammatory" OR "Antiinflammatories" OR "Anti inflammatories" OR "NonSteroidal" OR "Non Steroidal" OR "corticosteroid" OR "corticosteroid" OR "corticosteroids" OR "cortisone" OR "corticoid" OR "corticoids" OR "dexamethasone" OR "Methylfluorprednisolone" OR "Hexadecadrol" OR "Decameth" OR "Decaspray" OR "Dexasone" OR "Dexpak" OR "Maxidex" OR "Millicorten" OR "Oradexon" OR "Decaject" OR "Hexadrol" OR "Flubenisolone" OR "Betadexamethasone" OR "Celestona" OR "Celeston" OR "Celestone" OR "betamethasone" OR "flumethasone" OR "isoflupredone" OR "fludrocortisone" OR "triamcinolone" OR

"Volon" OR "Aristocort" OR "prednisolone" OR "Predate" OR "Predonine" OR "Di Adreson F" OR "betamethasone" OR hydrocortisone OR "Glucocorticoids" OR "Glucocorticoid" OR "Anticoagulants" OR "Anti coagulants" OR "Anticoagulant" OR "Anti coagulant" OR "Anticoagulation" OR "Anti coagulation" OR "Indirect Thrombin" OR "Anti thrombins" OR "Antithrombins" OR "drugs" OR "drug"

Concept 2:EHV1

"equine herpesvirus 1" OR "equine herpes virus 1" OR "Equine abortion Virus" OR "Equine abortion Viruses" OR "EHV 1" OR "EHV1" OR "equid herpesvirus 1" OR "equid herpesvirus type 1" OR "Equine herpesvirus myeloencephalopathy" OR "Equine herpes myeloencephalopathy" OR "EHM" OR "equine herpesvirus type 1" OR "equine herpes virus type 1" OR "equid alphaherpesvirus 1" OR "Equine alphaherpesvirus 1"

Concept 3: Horses

"horse" OR "horses" OR "equid" OR "equine" OR "equus"

Cochrane = 2

"pharmacology" OR "pharmacological" OR "pharmacologic" OR "Antiviral" OR "Anti Infective" OR "NSAIDs" OR "NSAID" OR "Anti Inflammatory" OR "AntiInflammatory" OR "Antiinflammatories" OR "Anti inflammatories" OR "NonSteroidal" OR "Non Steroidal" OR "corticosteroid" OR "corticosteroid" OR "corticosteroids" OR "cortisone" OR "corticoid" OR "corticoids" OR "dexamethasone" OR "Methylfluorprednisolone" OR "Hexadecadrol" OR "Decameth" OR "Decaspray" OR "Dexasone" OR "Dexpak" OR "Maxidex" OR "Millicorten" OR "Oradexon" OR "Decaject" OR "Hexadrol" OR "Flubenisolone" OR "Betadexamethasone" OR "Celestona" OR "Celeston" OR "Celestone" OR "betamethasone" OR "flumethasone" OR "isoflupredone" OR "fludrocortisone" OR "triamcinolone" OR "Volon" OR "Aristocort" OR "prednisolone" OR "Predate" OR "Predonine" OR "Di Adreson F" OR "betamethasone" OR hydrocortisone OR "Glucocorticoids" OR "Glucocorticoid" OR "Anticoagulants" OR "Anti coagulants" OR "Anticoagulant" OR "Anti coagulant" OR "Anticoagulation" OR "Anti coagulation" OR "Indirect Thrombin" OR "Anti thrombins" OR "Antithrombins" OR "drugs" OR "drug"

Concept 2:EHV1

"equine herpesvirus 1" OR "equine herpes virus 1" OR "Equine abortion Virus" OR "Equine abortion Viruses" OR "EHV 1" OR "EHV1" OR "equid herpesvirus 1" OR "equid herpesvirus type 1" OR "Equine herpesvirus myeloencephalopathy" OR "Equine herpes myeloencephalopathy" OR "EHM" OR "equine herpesvirus type 1" OR "equine herpes virus type 1" OR "equid alphaherpesvirus 1" OR "Equine alphaherpesvirus 1"

Concept 3: Horses

"horse" OR "horses" OR "equid" OR "equine" OR "equus"

Supplementary Item 3: List of studies that were excluded based on a review of the full text. The reason for exclusion is also provided.

Citation	Reason for exclusion
Anonymous. Tierarztl Prax Ausg G Grosstiere Nutztiere Feb 2018;46(1):65 2018 Feb	No original data
Anonymous. Tierarztl Prax Ausg G Grosstiere Nutztiere Feb 2018;46(1):65 2018 Feb	Duplicate record
British Equine Veterinary Association Congress 2013, Manchester, United Kingdom, 11-14 September 2013. Equine Vet J 2013;45():i-19	No original data
Cohen JC, Perdue ML, Randall CC, O'Callaghan DJ. Herpesvirus transcription: altered regulation induced by FUdR. Virology. 1977 Feb;76(2):621-33.	Wrong virus or no viral challenge
Cohen JC, Perdue ML, Randall CC, O'Callaghan DJ. Replication of equine herpesvirus type I: resistance to hydroxyurea. Virology. 1975 Sep;67(1):56-67.	Wrong virus or no viral challenge
Collins P. The spectrum of antiviral activities of acyclovir in vitro and in vivo. J Antimicrob Chemother. 1983 Sep;12 Suppl B:19-27.	Wrong animal species
Croubels,S. A pharmacokinetic and pharmacodynamic approach for dosing Valacyclovir against equine Herpesvirus type 1 infections. Pferdeheilkunde 2009;25(6):597-597	No original data
De Clercq E. The antiviral spectrum of (E)-5-(2-bromovinyl)-2'-deoxyuridine. J Antimicrob Chemother. 1984 Aug;14 Suppl A:85-95.	Wrong virus or no viral challenge
Garré B, Gryspeerdt A, Croubels S, De Backer P, Nauwynck H. Evaluation of orally administered valacyclovir in experimentally EHV1-infected ponies. Vet Microbiol. 2009 Mar 30;135(3-4):214-21.	Duplicate record
Gentry G, Allen G, McGowan J, Aswell J, Campbell D. Arabinosylthymine: in vivo effectiveness in a systemic herpesvirus infection. IARC Sci Publ. 1978;(24 Pt 2):1007-12.	Wrong animal species
Khurana SK, et al. Special Issue: Equine health, infectious diseases and zoonosis. J Exp Biol Ag Sci2016;4():S123-S210.	No original data
Kit S, Ichimura H, De Clercq E. Phosphorylation of nucleoside analogs by equine herpesvirus type 1 pyrimidine deoxyribonucleoside kinase. Antiviral Res. 1987 Jan;7(1):53-67.	Wrong virus or no viral challenge

Sakamoto K. Antiviral activity of 9-(2-hydroxyethoxymethyl) guanine against equine herpesvirus type 1 HH-1 strain and pseudorabies virus YS-81 strain. Nihon Juigaku Zasshi. 1990 Oct;52(5):1109-11.	Wrong animal species
Traub-Dargatz JL, Pelzel-McCluskey AM, Creekmore LH, Geiser-Novotny S, Kasari TR, Wiedenheft AM, Bush EJ, Bjork KE. Case-control study of a multistate equine herpesvirus myeloencephalopathy outbreak. J Vet Intern Med. 2013 Mar-Apr;27(2):339-46.	Wrong study design
Walter J, Seeh C, Fey K, Bleul U, Osterrieder N. Prevention of equine herpesvirus myeloencephalopathy - Is heparin a novel option? A case report. Tierarztl Prax Ausg G Grosstiere Nutztiere. 2016 Oct 12;44(5):313-317.	Duplicate record
Ziebell KL, Steinmann H, Kretzdorn D, Schlapp T, Failing K, Schmeer N. The use of Baypamun N in crowding associated infectious respiratory disease: efficacy of Baypamun N (freeze dried product) in 4-10 month old horses. Zentralbl Veterinarmed B. 1997 Nov;44(9):529-36.	Wrong virus or no viral challenge

Supplementary Item 4: List of included studies and funding source

Included Study	Funding Source
Brosnahan MM, Damiani A, van de Walle G, Erb H, Perkins GA, Osterrieder N. The effect of siRNA treatment on experimental equine herpesvirus type 1 (EHV-1) infection in horses. Virus Res. 2010 Feb;147(2):176-81.	Harry M. Zweig Memorial Fund for Equine Research at Cornell University; NIH training grant
Garré B, Gryspeerdt A, Croubels S, De Backer P, Nauwynck H. Evaluation of orally administered valacyclovir in experimentally EHV1-infected ponies. Vet Microbiol. 2009 Mar 30;135(3-4):214-21.	Not identified
Gibson JS, Slater JD, Field HJ. The activity of (S)-1-[(3-hydroxy-2-phosphonyl methoxy) propyl] cytosine (HPMPC) against equine herpesvirus-1 (EHV-1) in cell cultures, mice and horses. Antiviral Res. 1992 Sep;19(3):219-32.	Equine Virology Research Foundation. Agricultural and Food Research Council of Great Britain.
Maxwell LK, Bentz BG, Gilliam LL, Ritchey JW, Pusterla N, Eberle R, Holbrook TC, McFarlane D, Rezabek GB, Meinkoth J, Whitfield C, Goad CL, Allen GP. Efficacy of the early administration of valacyclovir hydrochloride for the treatment of neuropathogenic equine herpesvirus type-1 infection in horses. Am J Vet Res. 2017 Oct;78(10):1126-1139.	Grayson Jockey Club, Hagyard Equine Medical Institute, and NIH Short-Term Research Training grant
Ons E, Van Brussel L, Lane S, King V, Cullinane A, Kenna R, Lyons P, Hammond TA, Salt J, Raue R. Efficacy of a Parapoxvirus ovis-based immunomodulator against equine herpesvirus type 1 and Streptococcus equi equi infections in horses. Vet Microbiol. 2014 Oct 10;173(3-4):232-40.	Zoetis
Perkins GA, Van de Walle GR, Pusterla N, Erb HN, Osterrieder N. Evaluation of metaphylactic RNA interference to prevent equine herpesvirus type 1 infection in experimental herpesvirus myeloencephalopathy in horses. Am J Vet Res. 2013 Feb;74(2):248-56.	Harry M. Zweig Memorial Fund for Equine Research
Seahorn TL, Carter GK, Martens JG, Crandell RA, Martin MT, Scrutchfield WL, Cummins JM, Martens RJ. Effects of human alpha interferon on experimentally induced equine herpesvirus-1 infection in horses. Am J Vet Res. 1990 Dec;51(12):2006-10.	Not identified
Verma S; Tewari SC, Yadav MP. Use of polyherbal immunomodulator in conjunction with inactivated vaccine against equine herpes virus-1. Indian J Virol. 1999;15(1):63-67.	Not identified
Walter J, Seeh C, Fey K, Bleul U, Osterrieder N. Prevention of equine herpesvirus myeloencephalopathy - Is heparin a novel option? A case report. Tierarztl Prax Ausg G Grosstiere Nutztiere. 2016 Oct 12;44(5):313-317.	Not identified

Supplementary Item 5: Demographics and pre-study EHV-1 status of included studies

Study	Breed	Sex and number	Age range	Pre-study EHV-1 status
Brosnahan et al., 2010	Multiple	CM (n = 8); F (n = 8)	3 to 18 y	Negative antibody titer (SN ≤ 1:24)
Garré et al., 2009	Shetland ponies	M (n = 6); F (n = 2)	≤ 8 mo to 2 y	Negative antibody titers (SN < 2, IPMA < 10)
Gibson et al., 1992	Welsh Mountain pony	NR n = 5)	3-4 mo	SPF (E HV-1, EHV-2 and EHV-4 negative)
Maxwell et al., 2017	Light horse breeds	F (n = 18)	> 20 y	Seronegative for anti-EHV-1 antibodies (ELISA)
Ons et al., 2014	Gypsy Cob	M (n = 29)	6-8 mo	Negative antibody titer (SN ≤ 10)<br>Negative NP swab sample (PCR)
Perkins et al., 2013	NR	CM (n = 6); F (n = 7)	3-20 y	Negative antibody titer (SN ≤ 64)
Seahorn et al., 1990	Thoroughbred and Quarter horse	NR (n = 18)	5-7 months	Negative antibody titer (≤ 1:8)
Verma et al., 1999	Kathiawari	NR (n = 6)	NR	Negative antibody titer (SN = 2 [GM])
Walter et al., 2016	NR	M (n = 8); CM (n = 20); F (n = 33)	≤ 15 y	Negative antibody titer (SN ≤ 64)

Abbreviations: CM: castrated male (gelding); ELISA: enzyme-linked immunosorbent assay; F: female; GM: geometric mean; IPMA: immunoperoxidase monolayer assay; M: male (stallion or NR); mo: month; NP: nasopharyngeal; NR: not reported; PBO: placebo (control); PCR: polymerase chain reaction; Rx: treatment group; SN: serum neutralization; SPF: specific pathogen free; y: year
